# Evidence from ClinicalTrials.gov on the growth of Digital Health Technologies in neurology trials

**DOI:** 10.1038/s41746-023-00767-1

**Published:** 2023-02-10

**Authors:** Lars Masanneck, Pauline Gieseler, William J. Gordon, Sven G. Meuth, Ariel D. Stern

**Affiliations:** 1 Department of Neurology, Medical Faculty University Hospital Düsseldorf, Düsseldorf, Germany; 2 Hasso Plattner Institute, University of Potsdam, Potsdam, Germany; 3 Division of General Internal Medicine and Primary Care, Brigham and Women’s Hospital, Boston, MA USA; 4 Mass General Brigham, Somerville, MA USA; 5 Department of Biomedical Informatics, Harvard Medical School, Boston, MA USA; 6 Harvard Business School, Boston, MA USA; 7 Harvard-MIT Center for Regulatory Science, Boston, MA USA

**Keywords:** Clinical trials, Neurology

## Abstract

Digital Health Technologies (DHTs) such as connected sensors offer particular promise for improving data collection and patient empowerment in neurology research and care. This study analyzed the recent evolution of the use of DHTs in trials registered on ClinicalTrials.gov for four chronic neurological disorders: epilepsy, multiple sclerosis, Alzheimer’s, and Parkinson’s disease. We document growth in the collection of both more established digital measures (e.g., motor function) and more novel digital measures (e.g., speech) over recent years, highlighting contexts of use and key trends.

The burden of neurological disorders is growing in the US and abroad, with neurological conditions representing the most frequent cause of disability worldwide^1^. The resulting consequences are considerable individual suffering and substantial societal healthcare costs^2,3^. R&D investments are needed to identify new therapies, but clinical research faces challenges including high costs, administrative hurdles, and challenging patient recruitment^4–6^. Furthermore, clinical trials and their associated follow-up visits burden patients and their relatives^7^, especially for those with highly disabling neurological diseases.

Against this backdrop, the use of digital tools, such as connected sensors, promises to make research more patient-centered and move clinical trials beyond single “snapshots” of disease status toward more continuous measurement of chronic disorders^8^. Connected digital products^9^ or Digital Health Technologies (DHTs)^10,11^ are software-containing, patient-focused, portable, and connected sensors of health-related measurements, which describe different products, including many wearables. DHT examples from current FDA guidance include spirometers with smart connectivity, consumer activity trackers, and mobile applications for patients to report outcomes^11^. Such connected sensors may improve both the quantity and quality of data collection during clinical trials and enable patient recruitment in a less burdensome and more patient-empowering remote setting^9^.

In neurological diseases such as Parkinson’s disease (PD), use of validated, reliable, and sensitive tools has already been shown to provide better data on the “real-life distribution of disease severity, as it fluctuates longitudinally” in early-stage patients^12,13^. DHTs can therefore facilitate new study designs^12^, improve the efficiency of clinical trials^14–16^, and contribute to tackling many of the challenges neurological research faces. As more researchers adopt such technologies, understanding the use and development of DHTs in neurological clinical trials will be valuable for investigators, practicing clinicians, and those designing patient care pathways. As such, this study documents trends in the use of DHTs in neurology research and highlights opportunities for both R&D activities and care delivery going forward.

We assessed studies registered on ClinicalTrials.gov for four exemplary chronic neurological disorders. Building on methods from Marra et al. (2020)^9^, we identified trial-indication pairs for clinical studies in epilepsy, the neuroinflammatory disease multiple sclerosis (MS), and the two neurodegenerative diseases, PD and Alzheimer’s disease (AD). We present quantitative and qualitative (categorical) analyses of the trials that used connected sensors to describe technology adoption in neurology research and outline underlying trends over the years 2010-2021, inclusive.

Of the 6763 trial-indication pairs for epilepsy, MS, AD, and PD considered, 503 trial-indication pairs were identified as using DHTs by our search algorithm. After manual verification, 441 trial-indication pairs, associated with 430 unique clinical trials, were determined to be relevant and included in the analysis sample (Supplementary Fig. 1). Analysis sample trial counts varied significantly across indications: most frequent were studies in PD (198, 44.9%), followed by MS (119, 27.0%), AD (87, 19.7%), and epilepsy (37, 8.4%).

We document growth in the use of DHTs in neurological clinical trials, with a compound annual growth rate of ~39% from 2010 – 2020, consistent with trends in DHT growth that have been documented in registered clinical studies more broadly^9^. Most analyzed trials were interventional, with DHT trials based on registries having started only in recent years and typically incorporating some kind of digital activity or speech tracking (Fig. 1).

**Fig. 1:**
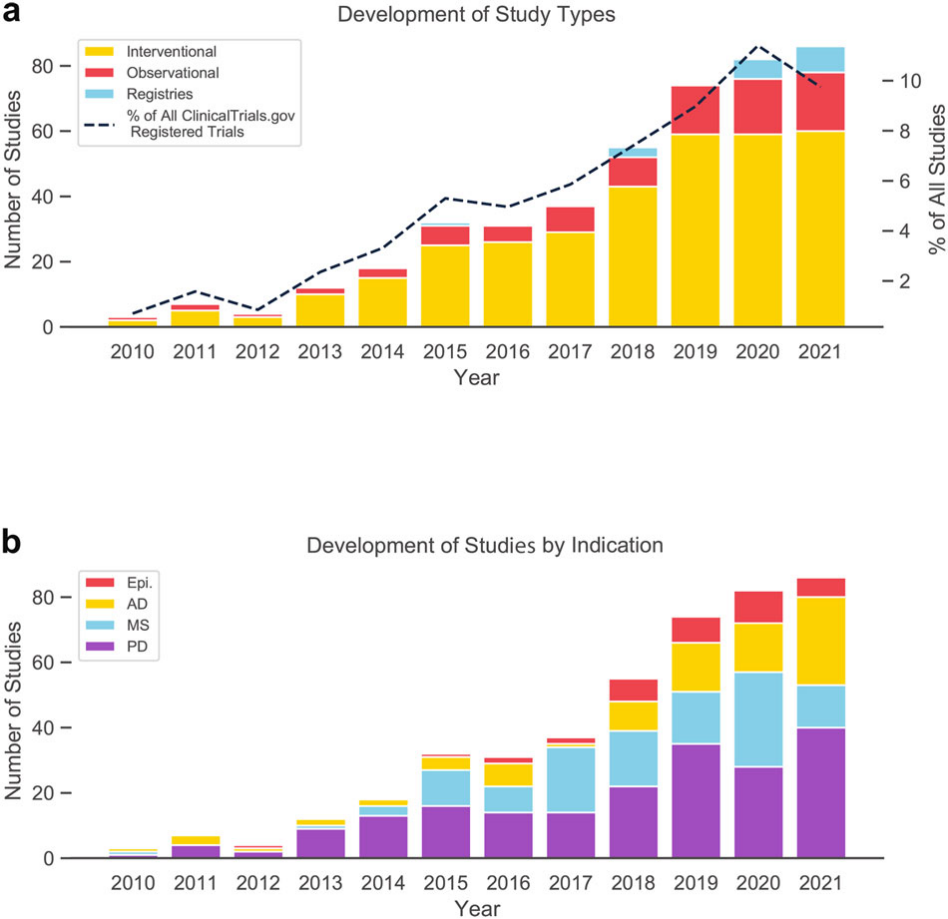
Development of clinical trials for chronic neurological diseases by year of initiation, trial type and indication. **a** Number of analyzed studies using Digital Health Technologies (DHTs) per year, stratified by the type of the respective trial. Line depicts the percentage of DHT trials out of all trials for the analyzed indications starting in the respective year. **b** Number of analyzed studies using DHTs per year, stratified by the indications epilepsy (Epi.), Alzheimer’s disease (AD), multiple sclerosis (MS), and Parkinson’s disease (PD). * Data for the year 2021 may be incomplete due to late registrations on ClinicalTrials.gov.

The relative frequency of DHT use in trials for the focal conditions increased from 0.7% of trials in 2010 to 11.4% in 2020. PD trials showed early uptake of DHTs and the highest use rate over the full period of the study (8.3% on average). AD, MS, and epilepsy trials showed a delayed upward trend, with respective use rates of 7.3%, 5.8%, and 3.3% overall (Supplementary Table 1).

Among all DHT trials, 16.6% included an industry sponsor or collaborator – slightly less frequently than the 19% previously documented across all medical disciplines^9^ – with industry partnerships more commonly observed in trials for epilepsy (37.8%) and AD (23.0%) (see Supplementary Fig. 2).

With respect to studies’ categorical (qualitative) features, most trials tracked patient symptoms (91.4%), with exceptions focusing on vital signs of caregivers or other disease-related outcomes such as medication adherence. The majority of trials measured motor functions (68.9%), with many including exercise elements (34.7%), sometimes through the use of gaming consoles (7.9%). Sleep (15.4%), cognition (10.4%), and speech (4.3%) tracking were less common as were gamification (19.3%), caregiver support (6.6%), and medication adherence (4.3%) elements (Table 1). A large share of studies used some form of mobile application (35.1%) and the most commonly referenced DHTs were wearables (129, 29.3%), smartphones (76, 17.2%), actigraphs (48, 10.9%) and mobile applications (42, 9.5%) (see Supplementary Table 2).

**Table 1:** Qualitative (categorical) features of trials by indication.

	All	Epilepsy	Alzheimer	Multiple Sclerosis	Parkinson
All Studies 2010-2021	6763	1131	1197	2062	2373
Eligible Studies 2010–2021 of all studies	441 (6.52% of 6763)	37 (3.27% of 1131)	87 (7.27% of 1197)	119 (5.77% of 2062)	198 (8.34% of 2373)
Industry-sponsored	90 (20.4% of 441)	14(37.84% of 37)	20 (22.99% of 87)	18 (15.13% of 119)	38 (19.19% of 198)
Gamification	85 (19.27% of 441)	2 (5.41% of 37)	14 (16.09% of 87)	31 (26.05% of 119)	38 (19.19% of 198)
Motor function tracking	304 (68.93% of 441)	15 (40.54% of 37)	34 (39.08% of 87)	92 (77.31% of 119)	163 (82.32% of 198)
Involves exercising	153 (34.69% of 441)	2 (5.41% of 37)	9 (10.34% of 87)	64 (53.78% of 199)	78 (39.39% of 198)
Involves exercising with gaming console	35 (7.93% of 441)	0 (0.00% of 37)	3 (3.45% of 87)	14 (11.76% of 119)	18 (9.09% of 198)
Tracking of disease symptoms	403 (91.38% of 441)	33 (89.19% of 37)	80 (91.95% of 87)	111 (93.28% of 119)	179 (90.40% of 198)
Sleep tracking	68 (15.42% of 441)	9 (24.31% of 37)	27 (31.03% of 87)	7 (5.88% of 119)	25 (12.62% of 198)
Speech tracking	19 (4.31% of 441)	1 (2.70% of 37)	11 (12.64% of 87)	0 (0.00% of 119)	7 (3.54% of 198)
Cognition tracking	46 (10.43% of 441)	0 (0.00% of 37)	16 (18.39% of 87)	18 (15.13% of 119)	12 (6.06% of 198)
Caregiver support	29 (6.58% of 441)	2 (5.41% of 37)	24 (27.69% of 87)	0 (0.00% of 119)	3 (1.52% of 198)
Medication adherence	19 (4.30% of 441)	6 (16.22% of 37)	0 (0.00% of 87)	5 (4.20% of 119)	8 (4.04 of 198)
Using phones	102 (23.13% of 441)	11 (29.73% of 37)	14 (16.09% of 87)	28 (23.53% of 119)	49 (24.74% of 198)
Using tablets	27 (6.12% of 441)	1 (2.70% of 37)	14 (16.09% of 87)	5 (4.20% of 119)	7 (3.54% of 198)
Using phones/ tablets/ mobile application	152 (34.47% of 441)	16 (43.24% of 37)	33 (37.93% of 87)	44 (36.97% of 119)	59 (29.80% of 198)

Shown are the number and percentage of trials meeting defined qualitative (categorical) features by indication. Percentages of trials applying Digital Health Technologies (DHTs) shown in the second row are calculated with respect to all trials for the indication. Percentages of study characteristics in other rows are calculated as a share of trials for that indication using DHTs.

PD and MS trials often focused on motor symptoms and physical exercise, with a high share of gamification elements in MS trials. Other observed characteristics included numerous caregiver support and speech recognition tools in AD trials, frequent sleep tracking in AD and epilepsy trials, cognition tracking in AD and MS trials, and a recurrent tracking of medication adherence in epilepsy trials (Table 1).

Generally, a trend toward more complex and more extensive studies was observed, as represented by a gradual change in categorical trial features over time (Fig. 2). Whereas the use of motor function and exercise tracking were already observed in 2010, speech and cognition tracking were not observed until the mid-2010s (Fig. 2). Speech and cognition tracking both grew over time and were present in 8.1% and 14.0% of analyzed DHTs using trials in 2021, respectively (Supplementary Table 1). Conversely, the relative use of gamification elements decreased slightly in more recent years (Supplementary Table 1).

**Fig. 2:**
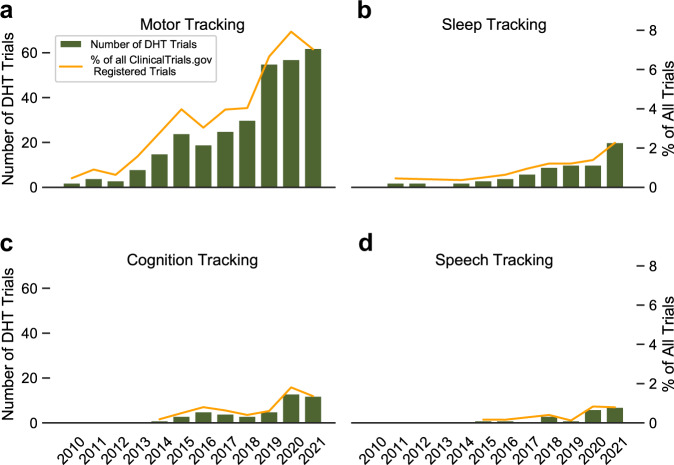
Use of selected Digital Health Technology tracking modalities in trials registered on ClinicalTrials.gov for chronic neurological diseases by year of trial initiation. Number of ClinicalTrials.gov registered trials of four different neurological indications using DHTs for different tracking modalities by trial initiation year (green bar plots). Additionally, rate of these studies compared to all trials for the same indications registered on ClinicalTrials.gov for the respective year is shown (orange line). Trials for the indications epilepsy, Alzheimer’s disease, multiple sclerosis, and Parkinson’s disease were included. **a** Absolute number and share of all trials for motor function tracking. **b** Absolute number and share of all trials for sleep tracking. **c** Absolute number and share of all trials for cognition tracking. **d** Absolute number and share of all trials for speech tracking. * Data for the year 2021 may be incomplete due to late registrations on clinicaltrials.gov.

Most registered trial-indication pairs were identified in North America or Europe, with a large share of trials having principal investigators in the US (209, 47.4%), followed by Italy and France (both 28 or 6.3% each), the UK (19, 4.3%), Belgium (17, 3.9%), Switzerland (16, 3.6%), Israel (14, 3.2%), Canada, Turkey, and Germany (all 13 or 2.9% each). In total, trials from 33 countries and all inhabited continents were included in the analysis sample. Some regions showed a local/regional concentration of DHT studies, often related to a specific indication. An interactive map displaying the locations of sample trials are publicly accessible at https://www.reine-nervensache.de/eigene-forschungsergebnisse/digital-health-technologies-in-neurology-trials.

Qualitatively, included studies displayed heterogeneity in their aims. Whereas some clinical trials aimed to validate products or novel biomarkers, others already applied them as endpoints in studies of other interventions.

While some have highlighted insufficient prospective clinical research applying DHTs and mobile health^17^, the use of DHTs in neurology trials has not previously been characterized in detail. We analyzed disease-specific DHT adoption in trials registered on ClinicalTrials.gov to provide insights specific to four chronic neurologic diseases.

The analysis confirmed a clear trend in DHT adoption, with some disease-specific heterogeneity. The earlier application of such tools in disorders with primarily motoric symptoms, such as PD or MS, is likely due to the early availability of wearable sensors to track motor functions and might contribute to the higher number of trials observed for these two indications. However, as novel sensors and sophisticated technical setups became available in more recent years, multimodal clinical trials tackling diverse research questions with DHTs began to emerge. This development is evident in the subset of AD trials, where recent growth in the share of trials using DHTs is consistent with increasing readiness of novel digital measures that integrate speech and cognition tracking^18,19^. In parallel, the repertoire of DHTs used in early-adopting subfields has also expanded, for example through potential PD monitoring technologies that now include passive tremor monitoring via wearables^12,13^, smartphone-based motor and vocal symptom assessments^20,21^, gait analysis via smart insoles^22^, and sensors for swallowing^23^.

Interestingly, pharmaceutical companies tested self-developed DHTs in some trials^24–26^, indicating a possibility for broader prospective adoption by other stakeholders. Differences in the share of industry-sponsored trials across neurologic diseases could indicate differences in experience with digital measures across indications or difference in research funding for different diseases (either within firms themselves and/or from investments by research foundations or public funders). Any of these factors could shape biopharmaceutical and medical device firms’ investments in their use in clinical trials. Regions with a higher density of clinical trials using DHTs differ in their indication-specificity, suggesting different backgrounds and foci of research teams: high-density areas with multiple indications were more frequent in the US and could indicate technology-driven research, whereas regional clusters focusing on research in a single indication appeared more common in other countries.

Some digital measures assessed in the analysis sample integrate information previously unavailable to clinicians and researchers, such as details of smartphone usage^27^ or characteristics of patient-caregiver-interactions^28,29^, in one case deploying real-time monitoring of mood and stress via a smart-home approach^29^. Monitoring cognitive and motor skills with ‘byproduct data’ such as metadata on smartphone keystroke dynamics, for example, in epilepsy^27^ or MS^30^, could allow remote monitoring to be passively and therefore more seamlessly integrated into patients’ daily lives and eventually, into clinical routines. Further examples of the use of previously inaccessible data for novel phenotyping include approaches such as longitudinal repetitive cognitive testing^18,31^, speech characterization^32^, or swallowing monitoring^23^. Especially in more complex recent studies, digital biomarkers or measures were observed in conjunction with biological and radiological biomarkers^22,33^, promising a more detailed overview of patients’ health status than ever before. Furthermore, the small but growing trend of monitoring and supporting caregivers or relatives via DHTs^28,29^ could preview new forms of promising assistance for these essential but often neglected alleviators of the burdens of chronic diseases. A personalized prevention of secondary burn-out or depression among caregivers could be supported by DHTs that objectively track interactions with patients and monitor caregivers’ vital signs and stress^28,29^.

The observed heterogenous use pattern of DHTs in the analysis sample highlights different use cases for validating DHTs, gathering data with DHTs, or using DHTs as interventions; understanding this heterogeneity should be a focus of future research. Examples of trials categorized by the use cases according to Marra et al.^9^ can be found in Supplementary Table 3. Generally, there appears to be a large variety of new therapeutic and monitoring solutions under study, although solutions often remain fragmented, stand-alone approaches, making it difficult to bring user-friendly, holistic approaches into practice at scale.

The growing share of disease-specific trials applying DHTs show that neurological research is already adapting and increasingly integrating remote digital measurements into its research agenda. In addition to growing adoption of established digital measures, such as motor function, novel approaches, such as speech and cognition tracking are being deployed more often.

Recent regulatory trends further support this development, as seen in the first qualification of a digital endpoint by the EMA in Duchenne muscular dystrophy^34^, a neuromuscular disorder, and published guidance for applying DHTs by the FDA^11^. Nevertheless, there remains ample opportunity to extend the use of DHTs in clinical trials and support their transition into routine clinical care. Different technologies promise improved patient healthcare access and new setups for decentralized clinical trials, thereby enabling previously unimaginable measures for gathering real-world evidence. Further research is needed to clarify what is required for broader adoption in practice, thus enabling the translation of verified, analytically validated, and clinically validated tools^35^ into improved patient care.

## Methods

We identified DHTs in trials registered on ClinicalTrials.gov following Marra et al. (2020)^9^ and using an updated and curated version of the searched product list (see Supplementary Note 1 and GitHub repository in the Data Availability Statement). Additionally, trials were filtered for the conditions epilepsy, MS, AD, or PD, resulting in a dataset built around unique trial-indication pairs (only 10 trials had multiple indications). The analysis sample was restricted to trials launched from 2010–2021 (inclusive) that had at least begun recruitment. Data were retrieved on February 28th, 2022. The resulting set of records was analyzed for the use of DHTs and trials that referenced such products were included in the analysis and mapped to a list of relevant categories defined for this purpose. This list included non-mutually-exclusive features such as different tracking characteristics as listed in Table 1 and Supplementary Table 1 (mapping was carried out by L.M. and P.G.). For comparison, other contemporaneous trials for the same indications were pulled from ClinicalTrials.gov. All analyses were run using Python 3.8 (Python Software Foundation, Delaware, USA). An interactive map of the principal trial locations was generated using plotly version 5.4.0^36^ after geocoding addresses with geopy version 2.2.0^37^. The co-author team included a patient with one of the focal disorders.

### Reporting summary

Further information on research design is available in the Nature Research Reporting Summary linked to this article.

## Supplementary information

Reporting Summary

Supplementary Materials

## Data Availability

The data that support the findings of this study are publicly available at https://github.com/Entspannter/DHTs-in-neurology-trials. A visualization of the of the interactive map in the GitHub repository can be accessed at https://www.reine-nervensache.de/eigene-forschungsergebnisse/digital-health-technologies-in-neurology-trials/.

## Code availability

Code and instructions to reproduce the study are available in a public GitHub repository at https://github.com/Entspannter/DHTs-in-neurology-trials.
