# Supplementary material for: Evidence from ClinicalTrials.gov on the growth of Digital Health Technologies in neurology trials
Source: NPJ Digit Med. 2023 Feb 10;6:23. doi: 10.1038/s41746-023-00767-1 (PMC9918454; doi:10.1038/s41746-023-00767-1)
Supplement: Supplementary file 1 — Reporting Summary [file 41746_2023_767_MOESM1_ESM.pdf]

nature portfolio

Corresponding author(s): Ariel Dora Stern

Last updated by author(s): Jan 22, 2023

Reporting Summary

Nature Portfolio wishes to improve the reproducibility of the work that we publish. This form provides structure for consistency and transparency in reporting. For further information on Nature Portfolio policies, see our Editorial Policies and the Editorial Policy Checklist.

Statistics

For all statistical analyses, confirm that the following items are present in the figure legend, table legend, main text, or Methods section.

n/a	Confirmed	
☐	☒	The exact sample size (*n*) for each experimental group/condition, given as a discrete number and unit of measurement
☐	☒	A statement on whether measurements were taken from distinct samples or whether the same sample was measured repeatedly
☐	☒	The statistical test(s) used AND whether they are one- or two-sided<br>*Only common tests should be described solely by name; describe more complex techniques in the Methods section.*
☒	☐	A description of all covariates tested
☒	☐	A description of any assumptions or corrections, such as tests of normality and adjustment for multiple comparisons
☒	☐	A full description of the statistical parameters including central tendency (e.g. means) or other basic estimates (e.g. regression coefficient) AND variation (e.g. standard deviation) or associated estimates of uncertainty (e.g. confidence intervals)
☒	☐	For null hypothesis testing, the test statistic (e.g. *F*, *t*, *r*) with confidence intervals, effect sizes, degrees of freedom and *P* value noted<br>*Give P values as exact values whenever suitable.*
☒	☐	For Bayesian analysis, information on the choice of priors and Markov chain Monte Carlo settings
☒	☐	For hierarchical and complex designs, identification of the appropriate level for tests and full reporting of outcomes
☒	☐	Estimates of effect sizes (e.g. Cohen's *d*, Pearson's *r*), indicating how they were calculated

Our web collection on statistics for biologists contains articles on many of the points above.

Software and code

Policy information about availability of computer code

Data collection	Stata MP (Version 16.0) combined with Microsoft Excel and Python 3.8.13 using pandas version 1.3.4. Instructions for data collection can be accessed in the accompanying GitHub repository.
Data analysis	Python 3.8.13 plotly version 5.4.0 and geopy version 2.2.0. Code for data analysis can be accessed in the accompanying GitHub repository.

For manuscripts utilizing custom algorithms or software that are central to the research but not yet described in published literature, software must be made available to editors and reviewers. We strongly encourage code deposition in a community repository (e.g. GitHub). See the Nature Portfolio guidelines for submitting code & software for further information.

Data

Policy information about availability of data

All manuscripts must include a data availability statement. This statement should provide the following information, where applicable:

- Accession codes, unique identifiers, or web links for publicly available datasets
- A description of any restrictions on data availability
- For clinical datasets or third party data, please ensure that the statement adheres to our policy

The data that support the findings of this study are publicly available at https://github.com/Entspannter/DHTs-in-neurology-trials.

Human research participants

Policy information about studies involving human research participants and Sex and Gender in Research.

Reporting on sex and gender	No data regarding sex or gender was collected or analyzed.
Population characteristics	No single participants were recruited as we analyzed aggregated trial data.
Recruitment	No single participants were recruited as we analyzed aggregated trial data.
Ethics oversight	As we analyzed publicly available data this was not necessary.

Note that full information on the approval of the study protocol must also be provided in the manuscript.

Field-specific reporting

Please select the one below that is the best fit for your research. If you are not sure, read the appropriate sections before making your selection.

☐ Life sciences ☒ Behavioural & social sciences ☐ Ecological, evolutionary & environmental sciences

For a reference copy of the document with all sections, see nature.com/documents/nr-reporting-summary-flat.pdf

Behavioural & social sciences study design

All studies must disclose on these points even when the disclosure is negative.

Study description	Quantitative methods; additionally qualitative mapping of features by domain experts
Research sample	All clinical trails registered on ClinicalTrials.gov, launched from 2021, inclusive
Sampling strategy	Full population of trials were used (no sampling)
Data collection	Data were collected downloaded electronically from ClinicalTrials.gov. Product lists were collated from several sources accodring to the 'Methods' section in Marra etl al. https://www.nature.com/articles/s41746-020-0259-x. Data on clinical trials were collected from ClinicalTrials.gov. Feaute mapping was carried out electronically in Microsoft Excel by two researchers idenpendently (LM and PG).<br><br>Instructions for data collection can be accessed in the accompanying GitHub repository https://github.com/Entspannter/DHTs-in-neurology-trials.
Timing	2000-2021, inclusive
Data exclusions	No data were excluded from initial sampling. Analysis was performed on the subset of clinical trials that met the criteria outlined in the manuscript. The subset of trials meeting the criteria was defined by an algortihm and double-checked by two domain experts.
Non-participation	n/a
Randomization	N/a

Reporting for specific materials, systems and methods

We require information from authors about some types of materials, experimental systems and methods used in many studies. Here, indicate whether each material, system or method listed is relevant to your study. If you are not sure if a list item applies to your research, read the appropriate section before selecting a response.

Materials & experimental systems

n/a	Involved in the study	
☒	☐	Antibodies
☒	☐	Eukaryotic cell lines
☒	☐	Palaeontology and archaeology
☒	☐	Animals and other organisms
☒	☐	Clinical data
☒	☐	Dual use research of concern

Methods

n/a	Involved in the study	
☒	☐	ChIP-seq
☒	☐	Flow cytometry
☒	☐	MRI-based neuroimaging
