# Supplementary material for: Evidence from ClinicalTrials.gov on the growth of Digital Health Technologies in neurology trials
Source: NPJ Digit Med. 2023 Feb 10;6:23. doi: 10.1038/s41746-023-00767-1 (PMC9918454; doi:10.1038/s41746-023-00767-1)
Supplement: Supplementary file 2 — Supplementary Materials [file 41746_2023_767_MOESM2_ESM.pdf]

Supplementary Tables

Supplementary Table 1

	2010	2012	2014	2016	2018	2020	2021	Entire Period
**Number of eligible trial-indication pairs (% of all in respective year)**	3 (0.70 % of 431)	4 (0.85% of 472)	18 (3.32% of 542)	31 (4.96% of 625)	55 (7.40% of 743)	82 (11.40% of 719)	86 (9.64% of 882)	441 (6.52% of 6763)
**Of these epilepsy (% of all epilepsy pairs in year)**	0 (0.00% of 78)	1 (1.52% of 66)	0 (0.00% of 73)	2 (1.92% of 104)	7 (5.88% of 119)	10 (10.30% of 97)	6 (3.90% of 128)	37 (3.27% of 1131)
**Of these AD (% of all AD pairs in year)**	1 (0.85 % of 118)	1 (0.74% of 136)	2 (1.48% of 135)	7 (3.98% of 176)	9 (4.55% of 198)	15 (8.11% of 185)	27 (10.93% of 247)	87 (7.27% of 119)
**Of these MS (% of all MS pairs in year)**	1 (0.85 % of 117)	0 (0.00% of 133)	3 (1.88% of 160)	8 (5.59% of 143)	17 (8.95% of 190)	29 (14.95% of 194)	13 (5.80% of 224)	119 (5.77% of 2062)
**Of these PD (% of all PD pairs in year)**	1 (0.85% of 118)	2 (1.42% of 140)	13 (7.47% of 174)	14 (6.93% of 202)	22 (9.32% of 236)	28 (11.52% of 243)	40 (14.13% of 283)	198 (8.34% of 2373)
**Gamification (% of eligible trial-indication pairs)**	2 (66.67% of 3)	2 (25.00% of 4)	5 (27.78% of 18)	8 (26.67% of 30)	9 (16.36% of 55)	11 (13.58% of 81)	17 (19.77% of 86)	85 (19.3% of 441)
**Motor tracking (% of eligible trial-indication pairs)**	2 (66.67% of 3)	3 (75.00% of 4)	15 (83.33% of 18)	19 (63.33% of 30)	30 (54.54% of 55)	57 (70.37% of 81)	62 (72.09% of 86)	304 (68.9% of 441)
**Virtual exercising (% of eligible trial-indication pairs)**	2 (66.67 % of 3)	3 (25.00% of 4)	9 (50.00% of 18)	10 (33.33% of 30)	18 (32.73% of 55)	29 (35.80% of 81)	29 (33.72% of 86)	35 (7.9% of 441)
**Symptom tracking (% of eligible trial-indication pairs)**	2 (66.67% of 3)	4 (100% of 4)	16 (88.89% of 18)	24 (80.00% of 30)	52 (94.55 % of 55)	75 (92.59% of 81)	83 (96.51% of 86)	403 (91.4% of 441)
**Sleep tracking (% of all eligible trial-indication pairs)**	0 (0.00% of 3)	2 (50.00% of 4)	2 (11.11% of 18)	4 (13.33% of 30)	9 (16.36% of 55)	10 (12.35% of 81)	20 (23.26% of 86)	68 (15.4% of 441)
**Cognition tracking (% of eligible trial-indication pairs)**	0 (0.00% of 3)	0 (0.00% of 4)	1 (5.56% of 18)	5 (16.67% of 30)	3 (5.45% of 55)	13 (16.05% of 81)	12 (13.95% of 86)	46 (10.4% of 441)
**Speech tracking (% of all eligible trial-indication pairs)**	0 (0.00% of 3)	0 (0.00% of 4)	0 (0.00% of 18)	1 (3.33% of 30)	3 (5.45% of 55)	6 (7.41% of 81)	7 (8.14% of 86)	19 (4.3 % of 441)
**Caregiver support (% of all eligible trial-indication pairs)**	0 (0.00% of 3)	0 (0.00% of 4)	0 (0.00% of 18)	2 (6.67% of 30)	4 (7.27% of 55)	7 (8.64% of 81)	12 (13.95% of 86)	29 (6.6% of 441)
**Medication intake (% of eligible trial-indication pairs)**	0 (0.00% of 3)	0 (0.00% of 4)	1 (5.56% of 18)	2 (6.67% of 30)	1 (1.82% of 55)	1 (1.23% of 81)	3 (3.49% of 86)	19 (4.3% of 441)

Supplementary Table 1: Development of trials using DHTs and their features over time (previous page)

Shown are the number and percentage of eligible trials by year and disorder in two-year steps. Additionally, percentages of trial-indication pairs meeting categorical criteria for exemplary years are given. Data for the year 2021 may be incomplete due to late registrations on clinicaltrials.gov.

Supplementary Table 2: Most frequently matched search terms for trial identification (next page)

The table shows the number of matched trials for the 30 most frequently matched search terms. For these summary statistics, only manually verified trials were taken into account. The search terms shown here are a subset of the 85 search terms that produced matches.

Supplementary Table 2

Search Term	Number of Matching Studies
wearable	129
smartphone	76
actigraph	48
mobile_app	42
fitbit	27
ipad	27
kinect	25
mobile_device	21
kinesia	20
apdm	18
digital_biomarker	16
smart_phone	15
xbox	14
iphone	13
smartwatch	13
android	11
nintendo_wii	10
sleep_monitor	9
inertial_measurement_units	9
gt3x_	8
fitbit_charge	7
actiwatch	7
mhealth	7
g_walk	7
apple_watch	5
omron	5
empatica	5
pamsys	5
biosensics	5

Supplementary Table 3

Use Case (as identified by Marra et al)	Illustrating Example
**Trial validates DHT's functionality**	Trial that aims to identify and validate suitable digital biomarkers recorded with a Verily Study Watch to serve as outcomes in future clinical trials for Parkinson's disease. (NCT04985539)
**Trial tests DHT's clinical usability**	Trial monitoring multiple sclerosis patients using the autoinjectors with accompanying smartphone applications. The study collected different measurements regarding usage patterns, satisfaction with the applications, and adherence to the applications. (NCT03808142)
**Trial uses DHT to capture endpoint data for another intervention or data of interest in an observational study**	A longitudinal smartphone-based cognition assessment battery (Altoida's Digital Neuro Signature, previously Altoida Neuro Motor Index) was used to track cognitive changes as a secondary outcome in an interventional trial assessing the treatment of Alzheimer's Disease patients with autologous adipose-derived mesenchymal stem cells. (NCT04228666)
**Trial uses DHT as the primary intervention or a digital therapeutic**	Trial that tests the effect of a smartphone app aiming to increase medication adherence in epilepsy patients. (NCT02646631)

Supplementary Table 3: Examples of the use of DHT in the analyzed trials

The table gives examples of how DHTs were used in the analyzed trials. The table and the exemplary use cases are based on the categorization provided by Marra et al (2020).

Supplementary Figures

Supplementary Figure 1

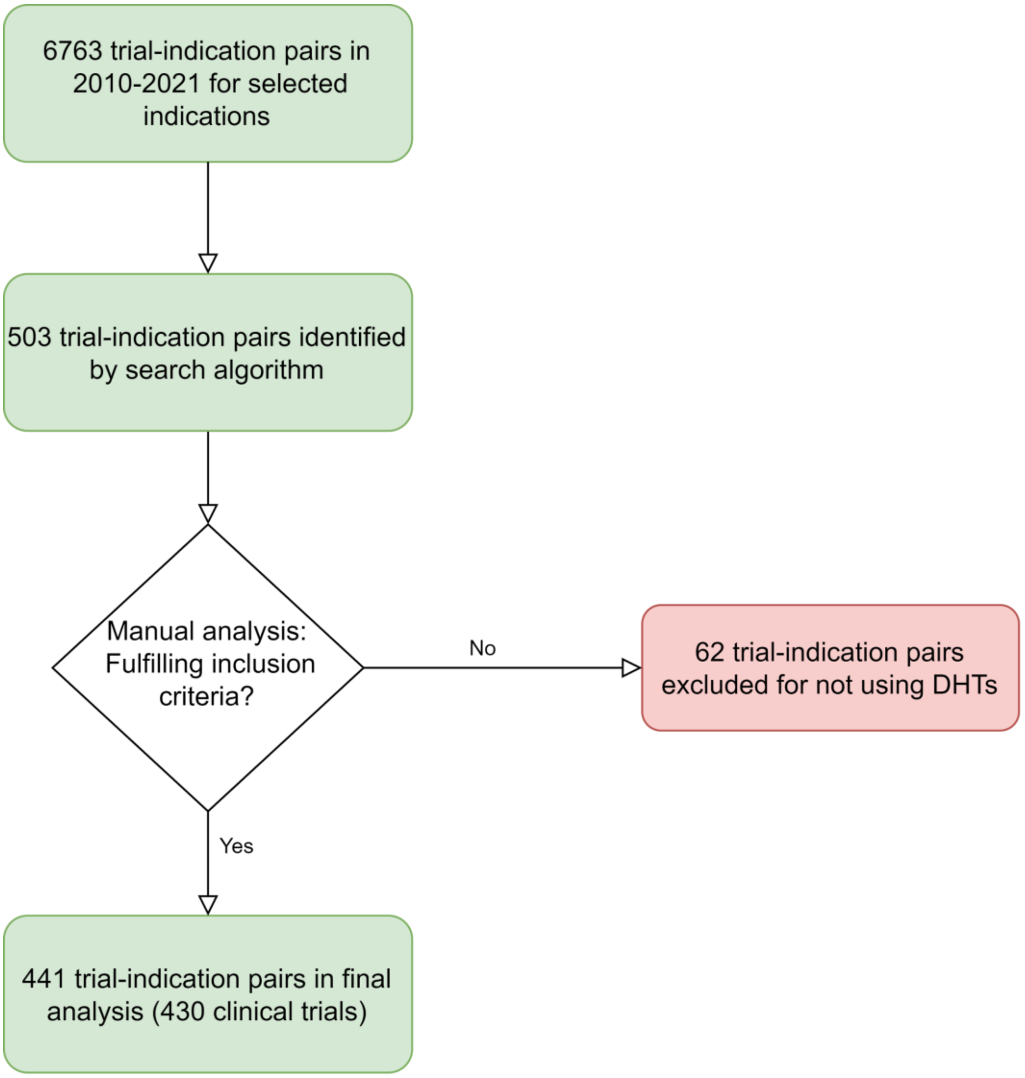

Supplementary Figure 1: Flow-chart of trial-indication pair selection process

Flow-chart of identifying suitable trial-indication pairs.

Supplementary Figure 2

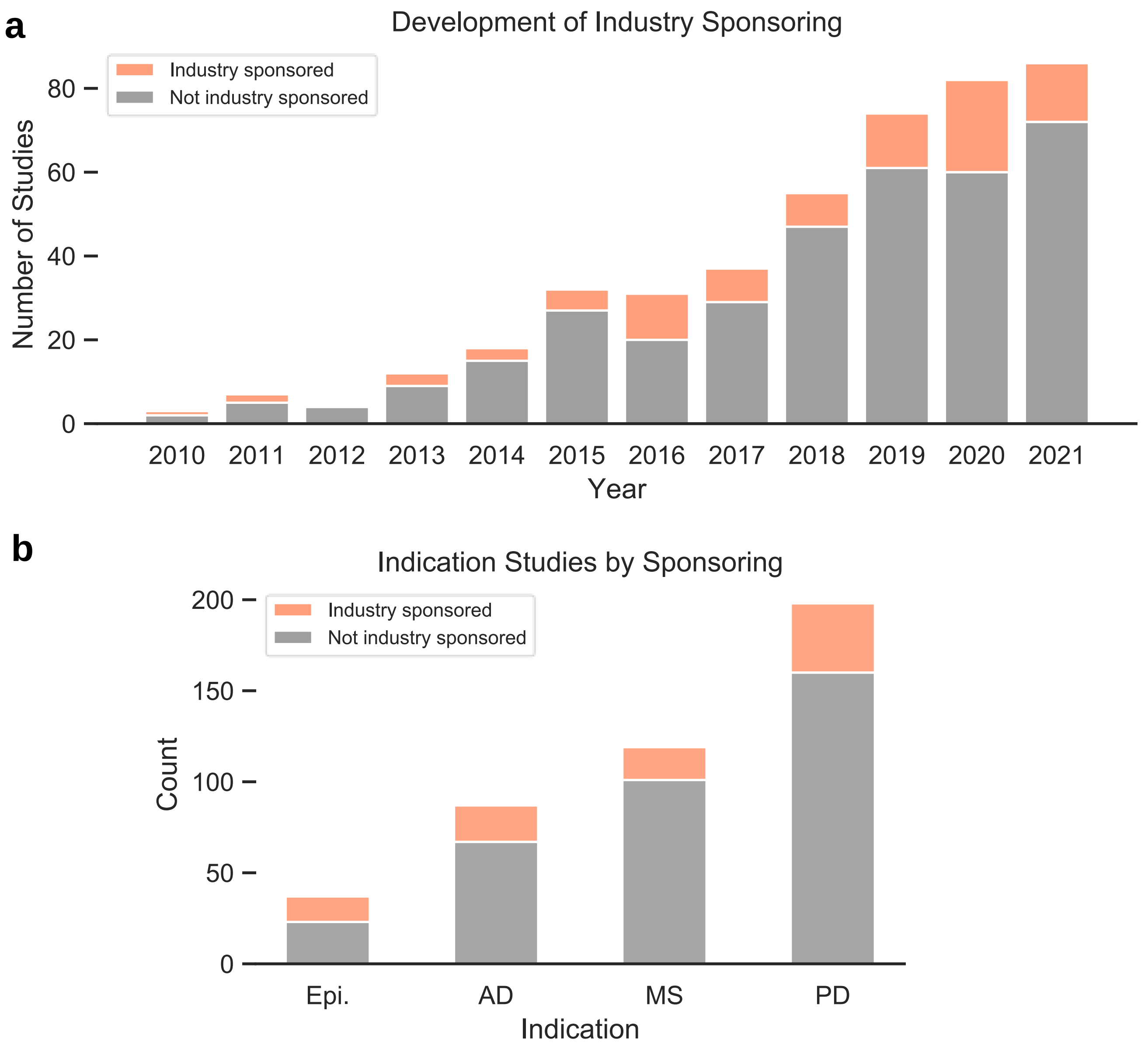

Supplementary Figure 2: Share of industry-sponsored studies by year of study start and indication

a. Number of analyzed trials using Digital Health Technologies per year of study start, stratified by industry sponsoring (upper orange bar indicates industry sponsored studies).

b. Absolute number of trials using Digital Health Technologies for each indication in the years 2010-2021, stratified by industry sponsoring (upper orange bar indicates industry sponsored studies). Analyzed disorders include epilepsy (**Epi**.), Alzheimer's disease (**AD**), multiple sclerosis (**MS**) and Parkinson’s Disease (**PD**).

Supplementary Notes

Supplementary Note 1

Within the special constraints of this manuscript it is not possible to describe in detail all the different DHTs used in the analyzed trials. There exist excellent databases open to researchers which match DHTs to indications and might serve as a good resource for further information. A prominent example of these is the Atlas by HumanFirst, which recently opened their database to academic researchers free of charge via the AtlasEDU program.

Files and instructions to reconstruct the used datasets are available at: https://github.com/Entspannter/DHTs-in-neurology-trials
